# Evaluating the Effect of an Educational Session on Nursing Faculty's Willingness to Serve as Mentors

**DOI:** 10.1155/jonm/7565000

**Published:** 2025-09-29

**Authors:** Emily J. Canale

**Affiliations:** Nursing Department, Arizona College of Nursing, Tempe, Arizona, USA

**Keywords:** educational intervention, faculty development, faculty retention, mentoring willingness, mentorship, nursing faculty, nursing shortage, preceptorship

## Abstract

The U.S. nursing shortage is exacerbated by high turnover among faculty and a lack of qualified mentors for novice nurses. Mentorship has been shown to improve clinical competency, faculty retention, and job satisfaction but remains underutilized due to barriers such as heavy workloads, mismatched work styles, and unclear expectations. This project assessed the impact of an educational session on nursing faculty's willingness to participate in mentoring relationships. A quasiexperimental, before-and-after design was used, with 39 faculty members from an accelerated nursing program in Arizona completing pre- and postintervention surveys. Data were analyzed using paired *t*-tests and the Wilcoxon signed-rank test. Results showed a statistically significant increase in willingness to mentor following the intervention (*M* = 45.26, SD = 4.115) compared to preintervention scores (*M* = 43.00, SD = 5.031), with a mean difference of 2.256 (*p*=0.009). A large effect size (*η*^2^ = 0.166) indicated a meaningful change in faculty perceptions. Normality tests confirmed the data met assumptions, and nonparametric analysis supported the results (*p*=0.013). These findings suggest that educational interventions highlighting the benefits of mentoring can significantly increase faculty's willingness to mentor. This could enhance faculty retention, improve student outcomes, and address the nursing shortage by promoting mentoring relationships in nursing education.

## 1. Introduction

The U.S. continues to face a severe nursing shortage, with cascading consequences for patient care and healthcare systems. In 2021, nursing schools turned away more than 90,000 qualified applicants due to insufficient faculty, classroom space, and clinical placements [1]. By 2022, more than 2000 full-time faculty positions remained vacant, representing a national vacancy rate of 8.8% [1]. This shortage threatens the profession's capacity to meet the Institute of Medicine's recommendation of 80% baccalaureate-prepared nurses, as only 65.2% of nurses currently hold a BSN or higher degree [1].

The National Council of State Boards of Nursing [2] projects that 20% of registered nurses will leave the workforce by 2027. Early-career nurses face particularly high attrition rates, with 35%–60% leaving their jobs within two years of graduation, often citing stress, burnout, and lack of transition support [3]. These workforce challenges intensify the need for faculty who can mentor and guide the next generation of nurses effectively. Mentorship has been consistently associated with higher confidence, reduced attrition, stronger teaching skills, and improved student outcomes [4].

The National League for Nursing [5] recognized mentorship as a critical strategy for faculty recruitment, retention, and succession planning [5]. Mentors, defined as experienced professionals guiding novices, provide psychosocial support, professional role modeling, and opportunities for personal growth [6]. In contrast, preceptors orient students in short-term clinical placements, often without formal preparation or long-term developmental focus [7]. Both models have value, but mentorship is distinct in its sustained emphasis on growth and career development. Despite evidence of its benefits, mentorship remains inconsistently implemented across academic and clinical settings, primarily due to a lack of time, resources, and institutional frameworks [8].

This project examined whether an educational intervention could enhance faculty willingness to mentor (WtM) , with the goal of contributing to workforce sustainability, improved student outcomes, and increased faculty retention.

### 1.1. History of Mentorships

Mentorship has been recognized as a powerful influence in personal and professional development for decades. Levinson [9] described mentors as pivotal figures who guide protégés into adulthood and the workforce by fostering development and modeling successful behaviors. This concept highlights the mentor's role as a trusted advisor who supports growth and goal achievement. In nursing, the NLN [5] emphasized mentorship as an essential practice to address anticipated faculty retirements and the urgent need to maintain educational capacity. The NLN argued that mentoring relationships are an indicator of excellence and called for structured, intentional mentoring processes. These historical insights reinforce mentorship as not merely supportive but as an obligation central to sustaining the nursing profession.

### 1.2. Models and Barriers of Mentorship

Preceptorship and mentorship, while related, serve different purposes. Preceptors are licensed nurses assigned to orient students or new staff within clinical units. Their focus is on short-term integration into routines, and they often lack formal preparation, contributing to variable student experiences [10]. In Ireland, Baxter and McGowan [11] found that students frequently felt invisible or unsupported during preceptorships due to staffing shortages and limited engagement. These findings echo those from the United States and Canada, where preceptors struggle with time pressures, competing priorities, and a lack of institutional clarity regarding expectations [12].

Mentorship extends beyond orientation, emphasizing developmental, reciprocal, and often long-term relationships. Mentoring can occur between faculty members, between students, or in peer-to-peer models and is consistently associated with increased confidence, competence, and professional identity [13]. Peer mentoring studies in Canada and the U.S. demonstrate mutual benefits for mentors and mentees, including reduced stress, enhanced performance, and increased confidence [12, 14]. Virtual mentorship programs have also been explored, with positive effects on academic socialization and mental well-being [15].

Despite these benefits, barriers to mentorship remain significant. Dahlke et al. [16] identified a lack of formal processes, limited mentor availability, and mismatched expectations as persistent obstacles. Wynn et al. [8] noted that organizational culture often fails to value mentorship, leaving faculty without incentives or recognition for their efforts. While global studies, such as Ughasoro et al. [17], reveal similar barriers in international contexts, nursing-specific research underscores the urgent need for structured mentorship frameworks in education and practice. Without political will, financial support, and institutional commitment, these initiatives risk remaining unsustainable.

### 1.3. Purpose

This project assessed whether a brief educational session could influence nursing faculty's WtM within an accelerated BSN program.

## 2. Methods

This project employed a quasiexperimental before-and-after design to measure changes in WtM. Participants were full-time faculty from an accelerated BSN program in Arizona, who were not currently engaged in mentoring relationships. Thirty-nine faculty members completed both pre- and postsurveys. Participant demographics are summarized in Table 1. A 30-min online educational session highlighted the benefits of mentorship and modeled effective mentoring behaviors, drawing on Bandura's social learning theory as the theoretical framework.

The WtM scale [18] was administered before and after the intervention. This validated 7-point Likert scale measures attitudes and intentions related to mentoring. Data were analyzed using paired *t*-tests and Wilcoxon signed-rank tests, with a significance set at *p* < 0.05. Effect sizes were calculated to determine the magnitude of change. IRB approval was obtained prior to participant recruitment. The paired *t*-test results are provided (see Table 2 for paired samples test results). Nonparametric analysis supported these findings (see Table 3 for Wilcoxon signed-rank test results).

### 2.1. Ethical Considerations

Before conducting this project, ethical issues and considerations were carefully reviewed. Voluntary participation, a key ethical principle, ensured that there was no coercion involved in the decision to participate in the project [19]. Participation in the research was both anonymous and voluntary. Protecting the anonymity of participants was essential, and all identifying information was either removed or disguised to safeguard their identities. There were no negative repercussions for individuals who chose not to participate in the study.

It was emphasized that any participant had the right to withdraw from the study at any time for any reason without facing any consequences [20]. The ethical standard of ensuring that participants were not placed in situations where they could be at risk of harm was followed, and no identifiable physical or psychological risks were identified as part of their participation in the research. Data collection was conducted transparently, and any limitations encountered throughout the research were documented.

## 3. Results and Discussion

**Figure 1:**
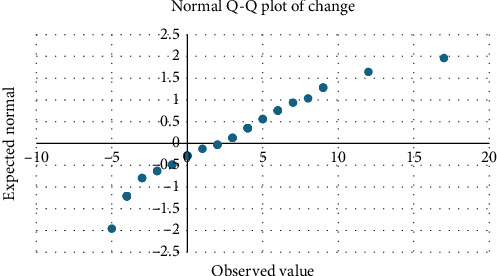
Q–Q plot of the change variable.

**Figure 2:**
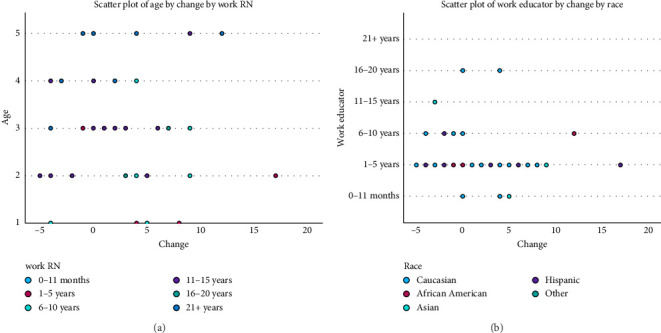
(a) Scatter plot of age by change by work RN. (b) Scatter plot of work educator by change by race.

The findings support the effectiveness of educational interventions in promoting mentorship engagement among nursing faculty. The statistically significant increase in WtM, supported by both parametric (Table 2) and nonparametric tests (Table 3), underscores the potential of structured sessions to address barriers such as lack of knowledge and undervaluing of mentorship roles. Normality testing (Table 4) and distribution analyses (Tables 5 and 6) confirmed the robustness of the results. Visual inspection further reinforced these findings: the Q-Q plot confirmed distributional assumptions (Figure 1), and the scatter plot highlighted demographic influences on change (Figure 2).

When effective, mentoring provides career and psychosocial support, including advising, sponsorship, and helping mentees develop a supportive network [21]. Despite this importance, especially in academics, mentorship rarely receives focused attention or evaluation in professional development such as teaching or research [21]. Opportunities exist to enhance the mentorship process, whether in formal or informal programs. Barriers exist to effective mentoring, such as a lack of mentor expertise, mismatched work styles and personalities, distancing behavior, and a lack of clear expectations and goals. Without political will and funding, mentoring initiatives may remain unsustainable.

Mentoring can help students and nurses in clinical competency and help reduce the chronic shortage of nursing staff [22]. Mentoring is critical during training to prepare nursing faculty and nursing students for their future roles as professional nurses. However, some professional nurses do not show the desired passion for mentoring others [22]. Schools of nursing and clinical partners need to advocate for state and federal funding to support protected mentoring time, stipends, and formal program development.

Despite all the positive aspects associated with mentorships, implementing mentoring is challenging [23]. A lack of standardization and shared views on mentoring, high workloads, organizational factors, and high turnover contribute to these challenges [23]. Identifying and understanding these barriers are crucial in nursing, as a lack of qualified nurses contributes to the overall patient safety in the United States healthcare system. Similar challenges have been documented in EU member states, where similar concerns have been discussed.

### 3.1. Major Findings

Postintervention scores increased significantly, rising from a mean of 43.00 (SD = 5.031) to 45.26 (SD = 4.115), *p* = 0.009 (Table 2). Normality testing (Table 4) and distribution analyses (Tables 5 and 6) confirmed the robustness of the results. Visual inspection further reinforced these findings: the Q-Q plot confirmed distributional assumptions (Figure 1), and the scatter plot highlighted demographic influences on change (Figure 2). The large effect size (*η*^2^ = 0.166) demonstrates meaningful improvement in faculty WtM (Table 2). Specific items revealed significant gains in participants' self-reported desire to participate in mentoring and their recognition of mentorship as an essential form of support. Nonsignificant findings included perceived time burden, though trends indicated participants viewed mentoring as less disruptive after the session. Relationships between demographic characteristics and change are further illustrated in the scatter plot of age and RN experience (Figure 1). These results suggest that even a brief intervention can positively influence faculty perceptions of mentoring.

### 3.2. Implications for Nursing Practice

Mentorship is a needed yet underutilized method for nursing students and faculty. Mentorship relationships play a key role in students' success, professional growth, and development [24]. Students can understand how to socialize in their roles when these relationships are effective. Barriers exist to mentoring, including the fact that most faculty do not receive formal mentorship education, and many institutions do not provide any requirements and/or incentives that communicate to faculty or students that mentorship is valuable [24]. Mentorship education could potentially improve effective mentorship practices and potentially promote a systematic change in the nursing field.

Nursing fields working with others may not always feel supported and may not have the educational background necessary to teach others, including mentoring [25]. Without proper guidance and support, they may not have the required competency for certain abilities, including giving prompt attention, setting goals, and/or developing critical thinking [25]. Understanding the role of being a mentor, including these expectations and goals, should be included in mentoring education [25]. Students believe conversing about their goals with mentors increases their learning capacity and action planning [25].

Educational sessions about mentorship could increase the willingness to participate and improve the outcomes of mentoring relationships. The American Nurses Association has developed a “Career Mentoring Resource,” where they provide advice from experienced members. They are promoting a flash mentoring program and a mentoring community page, where anyone in the organization can ask questions and engage in discussions [26]. Their career mentoring offers mentoring with an experienced nurse and a less experienced nurse for 6 months [26]. If one 30-min educational session impacts nurse faculty's willingness to participate in a mentoring relationship, a more formal mentoring educational program could increase nurses' WtM.

### 3.3. Recommendations

To diminish the nurse faculty shortage and better prepare nurses entering faculty roles, nursing schools should consider implementing methods such as mentorship programs [27]. When used effectively, they foster faculty development and address these shortages [27]. Mentorship programs are fundamental in guaranteeing adaptation at the healthcare level of new nurses, contribute to reducing the risk of mistakes, reduce stress, and have shown a reduction in the risks of clinical variability and attrition [28]. There is a wide range of mentoring programs with highly variable characteristics. The duration of these programs is also variable, with some ranging from eight weeks to others around 18 months [28].

Due to the variability in mentoring programs and recommendations, mentors may not have the necessary educational background, and research has suggested that mentorship systems being used may be ineffective [25]. This is partly due to variations in the mentor preparation programs and barriers to effective mentoring relationships [25]. Longitudinal mentorship has been shown to aid students in professional identity formation; however, over the past several decades, the clinical productivity demands on faculty have continued to increase [29]. This leads to mentoring relationships that are difficult to sustain [29]. To create effective mentorship programs and relationships, it is imperative that honoring a mentee's experiences and goals is facilitated, as well as establishing the mentor's role and expectations [29].

The American Nurses Association states that the success of mentoring will rely directly on the practicing organization's commitment to support the mentor role and the nurses who volunteer as mentors [30]. Therefore, it is imperative that the setting diligently works to provide support to the staff and builds a healthier work environment to promote mentorship [30]. It is suggested that a clear role is established when facilitating these standards, such as a mentor coordinator role, which has been proven to provide success in mentoring programs [30]. Distinct from precepting, mentoring is a tool that can leverage and create a sense of belonging and a pathway for professional growth [31]. It is recommended that a key strategy in successful mentorship is to develop the program within an established framework that entails a clear purpose and intent, a target audience, and specific outcomes [31]. The mentoring relationship should be personalized, where the mentor and mentee's needs, interests, and priorities are established [21].

### 3.4. Discussion

The findings support the effectiveness of educational interventions in promoting mentorship engagement among nursing faculty. The statistically significant increase in WtM underscores the potential of structured sessions to address barriers such as lack of knowledge, uncertainty about expectations, and undervaluing of mentorship roles. This aligns with the National Academies of Sciences [21], which emphasizes that mentorship education enhances the quality of relationships, improves communication, and supports alignment of expectations.

The implications extend beyond individual faculty development. Without political will and financial support, mentoring initiatives may remain underfunded, undervalued, and unsustainable. Nursing schools and clinical partners must advocate for policy support at the state and federal levels to secure resources such as protected mentoring time, stipends, and recognition structures. These measures are essential not only to faculty retention but also to ensuring consistent, high-quality mentorship for students. The findings also resonate with European contexts, where national health policies directly shape mentoring capacity and clinical placement structures [11]. Addressing mentorship as both an educational and political issue will strengthen its sustainability.

## 4. Conclusion

This project demonstrated that a single 30-min educational session can significantly improve faculty WtM. Mentorship must be recognized not only as a professional best practice but also as a strategic investment requiring political and financial support. By prioritizing mentorship, nursing programs can strengthen faculty development, support student success, and contribute to workforce sustainability at a time of critical shortage. Future research should expand these findings across diverse academic and clinical settings and explore the long-term impact of structured mentorship programs.

## Figures and Tables

**Table 1:** Demographic frequencies and percentages.

		**Frequency**	**Percent**

Age	18–29	4	10.3
	30–39	10	25.6
	40–49	14	35.9
	50–59	6	15.4
	60+	5	12.8

Gender	Female	36	92.3
	Male	3	7.7

Education	Masters	35	89.7
	Doctorate or PhD	4	10.3

Work RN experience	1–5 years	5	12.8
	6–10 years	10	25.6
	11–15 years	14	35.9
	16–20 years	2	5.1
	21+ years	8	20.5

Work educator experience	0–11 months	3	7.7
	1–5 years	27	69.2
	6–10 years	6	15.4
	11–15 years	1	2.6
	16–20 years	2	5.1

Race	Caucasian	27	69.2
	African American	3	7.7
	Asian	3	7.7
	Hispanic	6	15.4

**Table 2:** Paired samples test results.

**Paired samples statistics**									

	**Mean**			**N**		**Std. deviation**		**Std. error of the mean**	

Pair 1	TotalPost			45.26	39	4.115		0.659	
	TotalPre			43.00	39	5.031		0.806	

**Paired samples correlations**									

						**Significance**			

		**N**		**Correlation**		**One-sided *p***		**Two-sided *p***	

Pair 1	TotalPost and TotalPre	39		0.386		0.008		0.015	

**Paired Samples Test**									

**Pair**	**Mean**	**Std. deviation**	**Std. error of the mean**	**95% confidence interval of the difference (lower)**	**95% confidence interval of the difference (upper)**	**t**	**df**	**One-sided p**	**Two-sided p**

Pair 1: TotalPost–TotalPre	2.256	5.123	0.820	0.596	3.917	2.750	38	0.005	0.009

**Table 3:** Wilcoxon signed-rank test results.

	Null hypothesis	Test	Sig.^a,b^	Decision
1	The median of differences between TotalPre and TotalPost equals 0	Related samples Wilcoxon signed-rank test	0.013	Reject the null hypothesis

^a^The significance level is .050.

^b^Asymptotic significance is displayed.

**Table 4:** Normality testing results.

Tests of normality						
	Kolmogorov–Smirnov^a^			Shapiro–Wilk		
	Statistic	df	Sig.	Statistic	df	Sig.
Change	0.106	39	0.200^∗^	0.946	39	0.060

^a^Lilliefors significance correction.

^∗^This is a lower bound of the true significance.

**Table 5:** Distribution statistics.

			**Statistic**	**Std. error of the mean**

Change	Mean		2.26	0.820
	95% Confidence interval for the mean	Lower bound	0.60	
		Upper bound	3.92	
	5% Trimmed mean		1.95	
	Median		2.00	
	Variance		26.248	
	Std. deviation		5.123	
	Minimum		−5	
	Maximum		17	
	Range		22	
	Interquartile range		8	
	Skewness		0.651	0.378
	Kurtosis		0.294	0.741

**Table 6:** Paired samples details.

	Paired differences			95% confidence interval of the difference				Significance	
	Mean	Std. deviation	Std. error of the mean	Lower	Upper	*t*	df	One-sided *p*	Two-sided *p*
Pair 1 Q1-1–Q2-1	−0.744	2.185	0.350	−1.452	−0.035	−2.125	38	0.020	0.040
Pair 2 Q1-6–Q2-6	−0.385	1.042	0.167	−0.722	−0.047	−2.306	38	0.013	0.027
Pair 3 Q1-3–Q2-3	0.051	1.746	0.280	−0.515	0.617	0.183	38	0.428	0.855
Pair 4 TotalPre–TotalPost	−2.256	5.123	0.820	−3.917	−0.596	−2.750	38	0.005	0.009

## Data Availability

Data sharing is not applicable to this article as no new data were created or analyzed in this study.
